# Tracking the Selective Pressure Profile and Gene Flow of SARS-CoV-2 Delta Variant in Italy from April to October 2021 and Frequencies of Key Mutations from Three Representative Italian Regions

**DOI:** 10.3390/microorganisms11112644

**Published:** 2023-10-27

**Authors:** Alessandra Lo Presti, Angela Di Martino, Luigina Ambrosio, Luca De Sabato, Arnold Knijn, Gabriele Vaccari, Ilaria Di Bartolo, Stefano Morabito, Calogero Terregino, Alice Fusaro, Isabella Monne, Edoardo Giussani, Fabio Tramuto, Carmelo Massimo Maida, Walter Mazzucco, Claudio Costantino, Martina Rueca, Emanuela Giombini, Cesare Ernesto Maria Gruber, Maria Rosaria Capobianchi, Anna Teresa Palamara, Paola Stefanelli, on behalf of the Italian Genomic Laboratory Network

**Affiliations:** 1 Department of Infectious Diseases, Istituto Superiore di Sanità, 00161 Rome, Italy; angela.dimartino@iss.it (A.D.M.); luigina.ambrosio@iss.it (L.A.); annateresa.palamara@iss.it (A.T.P.); paola.stefanelli@iss.it (P.S.); 2 Department of Food Safety, Nutrition and Veterinary Public Health, Istituto Superiore di Sanità, 00161 Rome, Italy; luca.desabato@iss.it (L.D.S.); arnold.knijn@iss.it (A.K.); gabriele.vaccari@iss.it (G.V.); ilaria.dibartolo@iss.it (I.D.B.); stefano.morabito@iss.it (S.M.); 3 Division of Comparative Biomedical Sciences, Istituto Zooprofilattico Sperimentale delle Venezie, 35020 Padova, Italy; cterregino@izsvenezie.it (C.T.); afusaro@izsvenezie.it (A.F.); imonne@izsvenezie.it (I.M.); egiussani@izsvenezie.it (E.G.); 4 Clinical Epidemiology Unit and Regional Reference Laboratory, University Hospital “P. Giaccone”, 90127 Palermo, Italy; fabio.tramuto@unipa.it (F.T.); carmelo.maida@unipa.it (C.M.M.); walter.mazzucco@unipa.it (W.M.); claudio.costantino01@unipa.it (C.C.); 5 Department of Health Promotion, Mother and Child Care, Internal Medicine and Medical Specialties “G. D’Alessandro”, University of Palermo, 90127 Palermo, Italy; 6 Laboratory of Virology, National Institute for Infectious Diseases “Lazzaro Spallanzani” (IRCCS), 00149 Rome, Italy; martina.rueca@inmi.it (M.R.); dr.emanuela.giombini@gmail.com (E.G.); cesare.gruber@inmi.it (C.E.M.G.); mrcapobianchi@gmail.com (M.R.C.); 7 Saint Camillus International University of Health Sciences, Via di Sant’Alessandro, 8, 00131 Rome, Italy; 8 Department of Infectious Tropical Diseases and Microbiology, Sacro Cuore Don Calabria Hospital I.R.C.C.S., Via Don A. Sempreboni 5, 37024 Negrar di Valpolicella, Italy

**Keywords:** gene flows, mutations, SARS-CoV-2 Delta variant, selective pressure

## Abstract

The SARS-CoV-2 Delta variant of concern (VOC) was often associated with serious clinical course of the COVID-19 disease. Herein, we investigated the selective pressure, gene flow and evaluation on the frequencies of mutations causing amino acid substitutions in the Delta variant in three Italian regions. A total of 1500 SARS-CoV-2 Delta genomes, collected in Italy from April to October 2021 were investigated, including a subset of 596 from three Italian regions. The selective pressure and the frequency of amino acid substitutions and the prediction of their possible impact on the stability of the proteins were investigated. Delta variant dataset, in this study, identified 68 sites under positive selection: 16 in the spike (23.5%), 11 in nsp2 (16.2%) and 10 in nsp12 (14.7%) genes. Three of the positive sites in the spike were located in the receptor-binding domain (RBD). In Delta genomes from the three regions, 6 changes were identified as very common (>83.7%), 4 as common (>64.0%), 21 at low frequency (2.1%–25.0%) and 29 rare (≤2.0%). The detection of positive selection on key mutations may represent a model to identify recurrent signature mutations of the virus.

## 1. Introduction

The SARS-CoV-2 virus evolved rapidly with the emergence of new variants over time. Therefore, tracking the genome variability is essential to strengthen public health measures and preparedness, especially in the case of variants/mutations with possible impact on the transmissibility, severity and immunity [1,2]. The epidemiological consequences of novel mutations are closely related to their impact on viral replication, transmission and on the competition between co-circulating viral strains. According to the Pangolin classification, the Delta variant consisted of about 245 different sublineages AY.x in addition to the parental strain B.1.617.2 ([3], last access 5 October 2022). The SARS-CoV-2 Delta variant of concern (VOC) was dominant in Italy from mid-June until December 2021 [4,5]. Subsequently, the Delta variant has been de-escalated by the Centers for Disease Control and Prevention (CDC, April 2022) and the World Health Organization (WHO June 2022), due to the almost exclusive circulation of the Omicron variant. The European Centre for Disease Prevention and Control (ECDC) de-escalated BA.1 and BA.3 (Omicron) on 12 August 2022 [6]. At the time of writing, the BA.2, BA.4 and BA.5 (Omicron) were also de-escalated from the ECDC list of SARS-CoV-2 variants of concern (VOC) [7], as these lineages are no longer circulating in Europe.

Previous international studies provided genomic and selection assessment of SARS-CoV-2 Delta variant mainly grouped according to the continent (Europe, Asia, North America, South America, Africa, Oceania), [8] or to a specific country, i.e., India, the USA, Singapore, Israel [9,10]. Insights on SARS-CoV-2 lineage/sublineage classification, phylogeny, mutation identification and epidemiological features on genomes were reported at national level [11,12,13].

Herein, we investigated the gene flows and selective pressure by a bioinformatic approach on the Delta variant circulating in Italy in 2021, since this VOC was often associated with serious clinical course of the COVID-19 disease [14]. The frequency of key mutations localised in the positively selected sites identified in genomes from three representative Italian regions (Lazio, Sicily and Veneto) was also investigated.

Selective pressure is generally measured by the nonsynonymous/synonymous rate (dN/dS = ω), considering a nonsynonymous rate standing above the synonymous rate as evidence of selection [15]. Thus, when ω > 1 the amino acid (aa) change offers a selective advantage and is fixed at a faster rate than a synonymous mutation, evidencing a diversifying selection (positive selective pressure) [16]. Since the selective pressure profile of Delta variant in Italy remains poorly defined, this study can help to identify: (i) the positive and negative selection and the sites where they occur; (ii) the evolutionary dynamics and the recurrent mutations on those obtained from the three regions: Lazio, Sicily and Veneto; (iii) a pattern and compendium of mutations that need to be closely monitored, also on other future variants that will emerge [17], and stability of the proteins; (iv) a model to predict recurrent mutations.

## 2. Materials and Methods

### 2.1. Dataset and Sequence Alignment

A total of 1500 SARS-CoV-2 Delta variant genomes, collected in Italy from April to October 2021 (uploaded and analysed in the Italian COVID-19 Genomic I-Co-Gen national platform and deposited in GISAID) [18], were investigated. The dataset was built in relation to the total number of Delta genomes available at the 14 of October 2021. Specifically, 712 genomes from northern (47.4%), 259 from central (17.3%) and 529 (35.3%) from southern Italy were investigated. A total of 596 Delta genomes obtained from the above reported dataset were used to carry out an in-depth analysis, including sequences from three regions: north (Veneto), centre (Lazio) and south (Sicily) of Italy. These were used to estimate the genetic variability and the frequency of key mutations in the positively selected sites identified during the same study period.

For the purpose of the selective pressure analysis, the following protein-coding gene sequence subsets were defined: nsp1, nsp2, nsp3, nsp4, 3C-like proteinase (nsp5), nsp6, nsp7, nsp8, nsp9, nsp10, nsp11, nsp12, helicase (nsp13), 3′-to-5′-exonuclease (nsp14), endoRNAse (nsp15), 2′-O-ribosemethyltransferase (nsp16), S (surface glycoprotein), ORF3a, E, M, ORF6, ORF7a, ORF8, N and ORF10. All the sequence alignments were performed using the program MAFFT v.7 [19] with the Galaxy platform [20,21], followed by manual editing through the Bioedit program [22].

### 2.2. Gene Flow and Migration Analysis

The MacClade version 4 program (Sinauer Associates, Sunderland, MA) was used to test gene out/in flow in Italy, among SARS-CoV-2 Delta variant sequences, applying a modified version of the Slatkin and Maddison test [23]. A maximum likelihood (ML) phylogenetic tree was built using the IQ-TREE software v.1.6.12 [24] with the GTR model and used as the starting tree for this analysis. The ultrafast bootstrap approximation (UFBoot) and the SH-like approximate likelihood ratio test (SH-aLRT) were used for branch support values [25]. A one-character data matrix was obtained from the dataset by assigning to each taxon in the tree a one-letter code indicating its own sampling location, according to the different geographic areas in Italy (north, centre and south). The putative origin of each ancestral sequence (i.e., internal node) in the tree was inferred by finding the most parsimonious reconstruction (MPR) of the ancestral character. The final tree length, which is the number of observed gene flow events in the genealogy, can easily be computed and compared to the tree-length distribution of 10,000 trees obtained by random joining–splitting (null distribution). Observed genealogies significantly shorter than random trees indicated the presence of subdivided populations with restricted gene flow. The gene flow among the different geographic areas (character states) was traced with the state changes and stasis tool through the MacClade software [23], which counts the number of changes in a tree for each pairwise character state. When multiple MPRs were present, the algorithm calculated the average migration count over all possible MPRs for each pair.

### 2.3. Selective Pressure Analysis

The selective pressure analysis was performed on the above reported SARS-CoV-2 protein-coding sequence subsets, with the aim to characterise the SARS-CoV-2 variations and the evolutionary dynamics in Italy, identifying the statistically supported positive and negative selective pressure sites.

A positive diversifying selection was inferred on sites statistically significant for a value of nonsynonymous to synonymous substitution ω > 1, while purifying selection was inferred for ω < 1 [26]. On the contrary, neutrality was inferred for ω = 1 [26].

The fast unconstrained Bayesian approximation (FUBAR) and fixed effects likelihood (FEL) models were used [27,28] to identify selection under the HYPHY software v. 2.2.4 [29]. The FUBAR method infers the nonsynonymous (dN) and synonymous (dS) substitution rates on a per-site basis in large datasets, based on the assumption that a pervasive selection pressure is constant in the entire phylogeny [27].

The FEL model uses a ML approach to infer dN and dS substitution rates on a per-site basis for a given coding alignment and corresponding phylogeny [28]. This method assumes that the selection pressure for each site is constant along the entire phylogeny. 

Only the selective pressure sites confirmed by both FEL (*p* ≤ 0.05) and FUBAR (posterior probability ≥ 0.9) were reported as statistically supported.

The positions of the selective pressure sites and mutations in the different SARS-CoV-2 subsets were referred with respect to the protein products obtained from the SARS-CoV-2 reference Wuhan-Hu-1 (accession number: NC_045512.2).

The frequency of each amino acid substitution in the positively selected sites was calculated in the full dataset and in the subset of 596 SARS-CoV-2 Delta genomes from Lazio, Sicily and Veneto in order to classify them as very common, common, intermediate, at low frequency or rare. The prediction of the possible impact of the amino acid substitutions on the stability and structure of the protein was investigated through the I-Mutant 2.0 and PolyPhen-2 tools, respectively, as previously reported [30].

## 3. Results

### Gene Flow and Selective Pressure Analysis 

**Figure 1:**
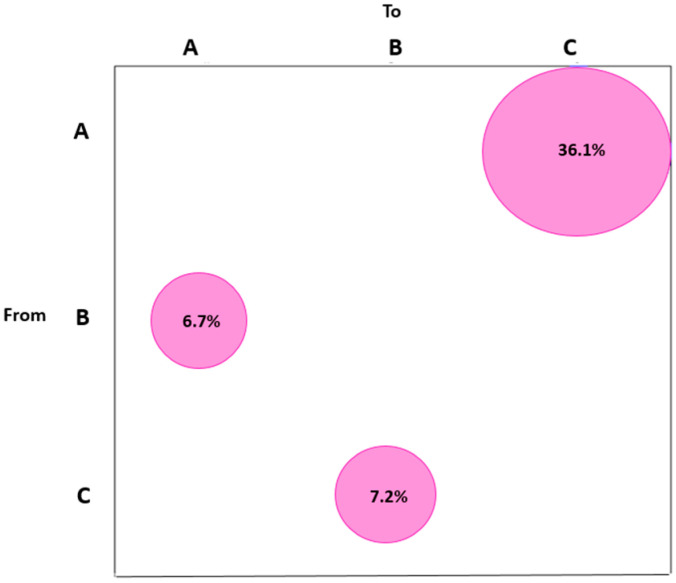
Maximum parsimony migration patterns of SARS-CoV-2 Delta variant genomes to/from different areas of Italy. The bubblegram shows the frequency of the statistically supported gene flow (migrations) identified to/from different geographic areas of Italy, as the percentage of the total observed migrations estimated from the maximum likelihood tree. A, north; B, centre; C, south.

The gene flow analysis performed according to the three geographic areas of Italy (north, centre and south), showed that most of the statistically supported gene flow events (36.1%) were identified from the north to the south; 6.7% of the supported gene flow events were found from the centre to the north; finally, 7.2% of supported gene flow was highlighted from the south to the centre of Italy (Figure 1).

Overall the selective pressure showed considerable variation among the SARS-CoV-2 protein coding genes. The analysis of the Delta variant Italian dataset revealed 68 positively selected sites dispersed in the different protein coding genes, as shown in Table S1. More than 9 positively selected sites were identified in nsp2, nsp12 and spike (Table S1). In particular, 11 positively selected sites (16.2%) were identified in nsp2, 10 in nsp12 (14.7%) and 12 in the spike (17.6%).

Among the positively selected sites identified inside the spike protein, three (367, 452 and 501) were located inside the RBD portion. Three to five positively selected sites were identified in nsp1, nsp3, nsp4, nsp6, nsp14 and nsp16 (Table S1). In detail, three sites were identified in nsp1 (4.4%), five in nsp3 (7.4%), four in nsp4 (5.9%), four in nsp6 (5.9%), four in nsp14 (5.9%) and three in nsp16 (4.4%). Few positively selected sites were identified in nsp13, nsp15, ORF3a, M and N protein coding genes (Table S1). No positively or negatively selected sites were identified in nsp11, in the envelope (E) or in ORF10 (Table S1). The analysis conducted on nsp5, nsp7, nsp8, nsp9, nsp10, ORF6, ORF7a and ORF8 indicated only negatively selected sites (Table S1).

The positively selected sites were further analysed to investigate the frequency of each amino acid replacement in our dataset (Table 1) in order to classify them as very common, common, at low frequency or rare. Six changes were identified as very common mutations (frequency > 83.7%), three substitutions were identified as common mutations (frequency > 64.0%), twenty-two mutations were identified at low frequency (between 2.1% and 25.0%) and fifty-three were rare (frequency ≤ 2.0%) (Table 1). Additionally, 85.7% of the amino acid replacements were predicted to decrease, 13.1% to increase and 1.2% not to change the stability of the protein (Table S2).

Overall, 29.8% of the amino acid changes were predicted by PolyPhen-2 as probably damaging the protein structure (score > 0.97), about 19.0% of the changes were predicted as possibly damaging, 48.8% as benign and, lastly, the probability of affecting protein structure was not known for 2.4% (Table S2).

The frequency of the key mutations in the positively selected sites in SARS-CoV-2 Delta genomes from Lazio, Sicily and Veneto altogether (Table 1) showed that 6 changes were identified as very common (frequency > 83.7%), 4 as common (frequency > 64.0%), 21 at low frequency (between 2.1% and 25.0%) and 29 were rare (frequency ≤ 2.0%) (Table 1). Twenty-four of the mutations in the positively selected sites previously reported in the full dataset (n = 1500) were not identified in the genomes from Lazio, Sicily and Veneto (n = 596).

The frequency estimated separately for each selected region showed in Lazio 9 changes as very common (frequency > 83.7%), no common mutations (frequency > 64.0%), 16 changes at low frequency (between 2.1% and 25.0%), 1 intermediate and 22 were identified as rare (frequency ≤ 2.0%). Thirty-six of the mutations in positively selected sites, reported for the full dataset, were not identified in the Delta genomes from Lazio (Table 1). In Sicily, 7 changes were identified as very common mutations (frequency > 83.7%), 3 substitutions as common (frequency > 64.0%), 14 were identified at low frequency (between 2.1% and 25.0%), 3 intermediate and 21 were rare (frequency ≤ 2.0%) (Table 1). Thirty-six of the mutations in positively selected sites, reported for the full dataset, were not identified in the genomes from Sicily. Finally, in Veneto, 7 changes were identified as very common mutations (frequency > 83.7%), 3 as common mutations (frequency > 64.0%), 16 were identified at low frequency (between 2.1% and 25.0%) and 13 were rare (frequency ≤ 2.0%). Forty-five of the mutations in positively selected sites, reported for the full dataset, were not identified in Veneto (Table 1).

Evident differences in frequencies of specific mutations were highlighted between genomes from Lazio, Sicily and Veneto (Table 1 and Table S3).

In particular, 10 mutations (K81N, E89K, S263F, A318V in nsp2; A46S, E61D in nsp12; G142D, A222V, Q613H in the spike; A110S in ORF3a) showed significantly higher frequency in Sicily respect to Lazio and Veneto (Table 1 and Table S3), 7 mutations (P1228L in nsp3; L838I in nsp12; A394V in nsp14; T95I, Q677H, D950N in the spike; Q9L in N) were significantly lower in Sicily respect to Lazio and Veneto (Table 1 and Table S3) and 2 mutations (Q822H in nsp12 and R289H in nsp14) showed significantly higher frequency in genomes from Lazio with respect to those from Sicily and Veneto (Table 1 and Table S3).

## 4. Discussion

The epidemic dynamics of COVID-19 in Italy and worldwide showed multiple waves, characterised by the emergence of different SARS-CoV-2 variants [2].

According to WHO data (COVID-19 Weekly Epidemiological Update) as of 30 March 2021, three variants were reported as emerging variants considered of concern (lineage B.1.1.7—Alpha variant, lineage B.1.351—Beta variant and lineage P.1—Gamma variant) [31]. Subsequently, the Delta variant (B.1.617.2 and AY.x lineages) was also classified as a “variant of concern” and became the dominant strain globally at that time.

Delta variant (B.1.617.2) emerged as the dominant across multiple countries and was endowed with enhanced infectivity and antibody escape capacity for the presence of key amino acid substitutions in the spike protein [32]. The Delta variant was associated with more severe infection, with patients more likely to be hospitalised and suffering longer infection course [33].

In Italy, Delta variant was dominant from mid-June until December 2021 [4,5]; afterward, Omicron variant became largely predominant [34,35]. This study provides a genomic analysis on Delta variant Italian dataset as a tool to identify the positive, negative selection, the evolutionary dynamics, and the recurrent mutations that need to be closely monitored also on other future variants for potential implications in public health.

The gene flow approach could help to identify the structure of the dispersal pattern and intermixing [23,36]. Overall, the study suggested that the gene flow of most of the SARS-CoV-2 Delta variant (36.1%) was from the northern to the southern part of Italy.

A similar percentage of gene flow (about 7.0%) was identified from central to northern of Italy and from southern to central.

The selective pressure analysis provided a large-scale genomic analysis towards understanding the selective pressure pattern on Italian Delta variant genomes. In addition, it allowed identification of the amino acid changes endowed of a selective advantage that were fixed at a faster rate than a synonymous mutation (positive selective pressure, ω > 1).

Most of the mutations identified in this study as positively selected sites, were also previously identified in other SARS-CoV-2 lineages internationally, as suggested by the genomes available in GISAID as of 20 October 2022 ([18], (last access 20 October 2022). 

In particular, already starting from the first epidemic phase, some of them (i.e., the mutations in the spike protein V367F, D614G [37] or the mutation A222V) emerged since summer 2020 in the 20E_EU1 cluster of the SARS-CoV-2 virus, presumably in Spain and then in Europe [38].

Most of the sites correlated with a greater pathogenicity, as for example the amino acid substitutions D614G, Q613H, N501Y, G142D, L452R or V367F (in the spike) [1,39,40].

The highest number of positive selected sites identified in the spike, followed by nsp2 and nsp12, suggested a possible evolutionary advantage to the virus, being specifically localised in regions or proteins with important functional roles (i.e., the receptor–binding domain RBD in the spike protein).

Three positive selected sites in RBD (amino acid positions 367, 452, 501) were found, likely conferring increased binding affinity for ACE2 [41].

The alterations in RBD, hypothesised as modifying RBD-ACE2 affinity, are generally rare [41]. Other authors suggested that the primary driver of positive selection arising from most mutations within the RBD is enhanced neutralisation resistance as opposed to increased affinity of S to ACE2 [41,42,43,44].

Some of the mutations detected at higher frequency in the full dataset were also confirmed at higher frequencies in genomes from the three representative areas (Lazio, Sicily, Veneto), such as G142D, L452R, D614G, P681R, D950N in the spike or the P323L in nsp12, probably indicating that these amino acid changes might favour viral adaptation.

An investigation of neutralising antibodies targeting the N-terminal domain (NTD) of the spike revealed a “supersite” for some known antibodies [45], considered a site of vulnerability for the SARS-CoV-2 virus. The T95I amino acid substitution does not occur close to the NTD neutralisation “supersite” but it was identified in our dataset as a positively selected site, with a frequency of about 20–21% among the sequences identified in Lazio, Veneto and about 12% in Sicily. A study performed on patients infected with SARS-CoV-2 showed an increased viral load for patients with variants showing the T95I [46]. Structural modelling analysis revealed that topological changes may occur in the NTD “supersite” as a result of the T95I, suggesting an effect of alteration of the topology of the supersite and affecting SARS-CoV-2 neutralisation by sera from vaccinated persons [41,46], suggesting the need to monitor all the mutations in the NTD region.

None of the positively selected sites identified in this study were already reported and included by COG.UK/Mutation Explorer in the list of the mutations conferring resistance to antiviral therapies ([47], last access 20 October 2022).

Six of the twenty-four mutations identified in the spike protein (T95I, G142D, A222V, V367F, L452R, N501Y) were associated with a weaker neutralisation of the virus by convalescent plasma from people who have been infected with SARS-CoV-2 and/or by monoclonal antibodies that recognise the SARS-CoV-2 spike protein (“escape” mutations) according to COG.UK [47]. Four of them were identified in the genomes from Lazio, Sicily and Veneto, and the N501Y only among sequences from Sicily.

Moreover, some of the amino acid changes were predicted to have a possible impact on the structure and stability of the proteins and need to be closely monitored. The detection of positive selection may represent an approach to identify key signature mutations.

Before drawing conclusions, limits and possible bias of the study have to be mentioned. This model is dependent on the availability of SARS-CoV-2 genomes - and on the limits imposed by the models used for the analysis.

The findings might provide a compendium of the SARS-CoV-2 mutations fixed at a faster rate, relative to synonymous changes and on the selective pressure profile in a Delta variant Italian dataset.

The selective pressure was probably the most likely reason for convergent evolution, that is different variants acquiring independently a group of recurrent mutations (i.e., residues K417, L452, E484, N501 and P681 of the spike for Alpha, Beta, Gamma and Delta variants or residues R346, K444, N450, N460, F486, F490, Q493 and S494 for Omicron and its sublineages) [47].

This study may update information on previous circulating SARS-CoV-2 strains, and help to track the presence of specific mutations in key viral genes.

## 5. Conclusions

In conclusion, this study provides a picture of the selective pressure profile and gene flows in a subset of Delta variant genomes identified in Italy, highlighting how specific mutations may become fixed in this viral population, how they affect the stability of the proteins, and, finally provides a model for recurrent mutations.

## Figures and Tables

**Table 1:** Frequency of amino acid mutations identified as positively selected sites from SARS-CoV-2 Delta genomes collected from the full dataset and from three regions (total number n = 1500 and n = 596, of which 213 are from Lazio, 245 from Sicily and 138 from Veneto).

Mutation	Target	% (n = 1500)	% (n = 596)	% (n = 213, Lazio)	% (n = 245, Sicily)	% (n = 138, Veneto)
P62S	nsp1	0.50%	0.70%	1.40%	0.40%	0.00%
E87D	nsp1	4.20%	5.20%	7.00%	5.30%	2.20%
G94S	nsp1	0.50%	1.20%	2.80%	0.00%	0.70%
G94V	nsp1	0.10%	0.20%	0.00%	0.40%	0.00%
R27C	nsp2	1.70%	0.00%	0.00%	0.00%	0.00%
K81N	nsp2	15.20%	24.20%	15.00%	36.70%	15.90%
E89K	nsp2	0.90%	2.30%	0.00%	5.70%	0.00%
P129L	nsp2	4.70%	3.40%	2.80%	2.90%	5.10%
P129S	nsp2	0.10%	0.00%	0.00%	0.00%	0.00%
D155G	nsp2	0.90%	0.80%	1.40%	0.80%	0.00%
A159V	nsp2	0.30%	0.00%	0.00%	0.00%	0.00%
S263F	nsp2	3.10%	6.00%	1.40%	13.50%	0.00%
A318V	nsp2	6.10%	11.90%	4.20%	21.60%	6.50%
G339S	nsp2	0.80%	1.30%	0.90%	1.60%	1.40%
V485I	nsp2	2.30%	1.30%	1.40%	0.80%	2.20%
Q496P	nsp2	1.20%	0.80%	0.90%	1.20%	0.00%
Q496H	nsp2	0.10%	0.00%	0.00%	0.00%	0.00%
S126L	nsp3	0.30%	0.20%	0.00%	0.00%	0.70%
K384N	nsp3	0.30%	0.30%	0.50%	0.00%	0.70%
L862F	nsp3	0.20%	0.00%	0.00%	0.00%	0.00%
P1228L	nsp3	74.10%	78.20%	88.30%	66.50%	83.30%
L1791F	nsp3	0.30%	0.20%	0.00%	0.00%	0.70%
T204I	nsp4	0.40%	0.70%	0.90%	0.40%	0.70%
D279N	nsp4	0.20%	0.00%	0.00%	0.00%	0.00%
T295I	nsp4	2.20%	5.00%	0.00%	12.20%	0.00%
C296F	nsp4	1.30%	0.70%	0.90%	0.40%	0.70%
A2V	nsp6	2.60%	2.30%	3.30%	0.00%	5.10%
T6I	nsp6	0.50%	1.20%	3.30%	0.00%	0.00%
Q27R	nsp6	0.50%	1.20%	0.00%	0.00%	5.10%
L37F	nsp6	2.10%	1.80%	4.20%	0.40%	0.70%
A46S	nsp12	2.30%	5.20%	0.00%	12.70%	0.00%
E61D	nsp12	1.40%	3.50%	0.00%	8.60%	0.00%
E61K	nsp12	0.10%	0.00%	0.00%	0.00%	0.00%
A95S	nsp12	0.50%	0.30%	0.90%	0.00%	0.00%
T141I	nsp12	0.50%	0.00%	0.00%	0.00%	0.00%
R197Q	nsp12	2.30%	3.00%	3.30%	0.80%	6.50%
P323L	nsp12	98.50%	97.30%	100.00%	93.50%	100.00%
S384P	nsp12	1.10%	0.80%	0.90%	1.20%	0.00%
M463I	nsp12	1.00%	0.80%	0.50%	1.60%	0.00%
Q822H	nsp12	3.10%	5.70%	13.60%	0.40%	2.90%
L838I	nsp12	12.80%	12.40%	16.00%	4.10%	21.70%
P77L	nsp13	95.90%	99.50%	100.00%	100.00%	97.80%
V89I	nsp13	0.30%	0.00%	0.00%	0.00%	0.00%
P46L	nsp14	2.10%	2.20%	1.90%	2.90%	1.40%
R289H	nsp14	3.00%	5.20%	12.70%	0.80%	1.40%
S374F	nsp14	0.40%	0.30%	0.50%	0.00%	0.70%
A394V	nsp14	69.00%	78.20%	88.30%	66.50%	83.30%
A80V	nsp15	0.50%	1.00%	0.00%	2.40%	0.00%
A81V	nsp15	0.70%	0.20%	0.50%	0.00%	0.00%
V9I	nsp16	0.40%	0.00%	0.00%	0.00%	0.00%
A34V	nsp16	0.50%	1.20%	0.00%	1.60%	2.20%
P215L	nsp16	0.10%	0.20%	0.50%	0.00%	0.00%
P215T	nsp16	0.70%	0.80%	0.90%	1.20%	0.00%
L5F	spike	0.90%	0.30%	0.50%	0.00%	0.70%
V70I	spike	0.10%	0.00%	0.00%	0.00%	0.00%
V70F	spike	0.10%	0.00%	0.00%	0.00%	0.00%
T95I	spike	20.90%	16.80%	19.70%	11.80%	21.00%
G142D	spike	64.10%	73.50%	55.40%	93.10%	66.70%
G142Y	spike	0.10%	0.00%	0.00%	0.00%	0.00%
G142H	spike	0.10%	0.00%	0.00%	0.00%	0.00%
G142V	spike	0.10%	0.00%	0.00%	0.00%	0.00%
A222V	spike	20.90%	19.00%	8.90%	30.60%	13.80%
A222S	spike	0.10%	0.00%	0.00%	0.00%	0.00%
V367L	spike	0.10%	0.00%	0.00%	0.00%	0.00%
V367H	spike	0.10%	0.00%	0.00%	0.00%	0.00%
V367F	spike	0.10%	0.00%	0.00%	0.00%	0.00%
L452R	spike	98.60%	98.00%	94.40%	100.00%	100.00%
Q613H	spike	6.60%	13.60%	1.90%	29.80%	2.90%
N501Y	spike	1.20%	0.20%	0.00%	0.40%	0.00%
D614G	spike	90.80%	100.00%	100.00%	100.00%	100.00%
Q677H	spike	3.80%	4.00%	5.60%	1.60%	5.80%
P681R	spike	93.90%	100.00%	100.00%	100.00%	100.00%
D950N	spike	83.70%	82.00%	91.50%	65.30%	97.10%
V1104L	spike	0.90%	0.70%	0.50%	0.40%	1.40%
V1128L	spike	1.70%	0.00%	0.00%	0.00%	0.00%
G1219V	spike	0.40%	0.20%	0.00%	0.40%	0.00%
G1219C	spike	0.20%	0.00%	0.00%	0.00%	0.00%
L41F	ORF3a	0.30%	0.30%	0.50%	0.40%	0.00%
L41I	ORF3a	0.10%	0.00%	0.00%	0.00%	0.00%
A110S	ORF3a	2.90%	6.00%	0.50%	14.30%	0.00%
A110V	ORF3a	0.10%	0.00%	0.00%	0.00%	0.00%
I82T	M	2.00%	100.00%	100.00%	100.00%	100.00%
Q9L	N	14.10%	12.10%	16.00%	4.10%	20.30%
Q9H	N	0.10%	0.00%	0.00%	0.00%	0.00%

## Data Availability

The SARS-CoV-2 sequences used in this study are available in GISAID (https://gisaid.org/ accessed on 14 October 2021).

## Supplementary Materials

The following supporting information can be downloaded at: https://www.mdpi.com/article/10.3390/microorganisms11112644/s1. Table S1: Selective pressure analysis of SARS-CoV-2 Delta variant Italian protein-coding subset genomes; Table S2: The prediction of the possible impact of the amino acid substitutions on the stability and structure of the proteins through I-Mutant 2.0 and PolyPhen-2; Table S3: Significance identified for each amino acid mutation by comparing data among the three regions.

## References

[1] European Centre for Disease Prevention and Control (ECDC) SARS-CoV-2 Variants of Concern as of 23 March 2023. https://www.ecdc.europa.eu/en/covid-19/variants-concern.

[2] Mahilkar S., Agrawal S., Chaudhary S., Parikh S., Sonkar S.C., Verma D.K., Chitalia V., Mehta D., Koner B.C., Vijay N. (2022). SARS-CoV-2 variants: Impact on biological and clinical outcome. Front. Med..

[3] Cov-Lineages.org–Lineage List. https://cov-lineages.org/lineage_list.html.

[4] Stima Della Prevalenza delle Varianti VOC (Variants of Concern) in Italia: Beta, Gamma, Delta, Omicron e Altre Varianti di SARS-CoV-2. Quick Survey 20 December 2021. https://www.epicentro.iss.it/coronavirus/pdf/sars-cov-2-monitoraggio-varianti-indagini-rapide-20-dicembre-2021.pdf.

[5] Prevalenza e Distribuzione Delle Varianti di SARS-CoV-2 di Interesse per la Sanità Pubblica in Italia Rapporto n. 15–10 December 2021. https://www.epicentro.iss.it/coronavirus/pdf/sars-cov-2-monitoraggio-varianti-rapporti-periodici-10-dicembre-2021.pdf.

[6] European Centre for Disease Prevention and Control Communicable Disease Threats Report, Week 32 7–13 August 2022. https://www.ecdc.europa.eu/sites/default/files/documents/Communicable-disease-threats-report-13-aug-2022-all-users.pdf.

[7] European Centre for Disease Prevention and Control ECDC de-Escalates BA.2, BA.4 and BA.5 from Its List of Variants of Concern. https://www.ecdc.europa.eu/en/news-events/ecdc-de-escalates-ba2-ba4-and-ba5-its-list-variants-concern.

[8] Middleton C., Kubatko L. (2023). Assessment of positive selection across SARS-CoV-2 variants via maximum likelihood. PLoS ONE.

[9] Zhang J., Fan L., Xu H., Fu Y., Peng X., Zheng Y., Yu J., He J. (2022). Evolutionary Pattern Comparisons of the SARS-CoV-2 Delta Variant in Countries/Regions with High and Low Vaccine Coverage. Viruses.

[10] Li K., Melnychuk S., Sandstrom P., Ji H. (2023). Tracking the evolution of the SARS-CoV-2 Delta variant of concern: Analysis of genetic diversity and selection across the whole viral genome. Front. Microbiol..

[11] De Marco C., Veneziano C., Massacci A., Pallocca M., Marascio N., Quirino A., Barreca G.S., Giancotti A., Gallo L., Lamberti A.G. (2022). Dynamics of Viral Infection and Evolution of SARS-CoV-2 Variants in the Calabria Area of Southern Italy. Front. Microbiol..

[12] Baj A., Novazzi F., Ferrante F.D., Genoni A., Tettamanzi E., Catanoso G. (2021). Spike protein evolution in the SARS-CoV-2 Delta variant of concern: A case series from Northern Lombardy. Emerg. Microbes Infect..

[13] Lai A., Bergna A., Della Ventura C., Menzo S., Bruzzone B., Sagradi F., Ceccherini-Silberstein F., Weisz A., Clementi N., Brindicci G. (2022). Epidemiological and Clinical Features of SARS-CoV-2 Variants Circulating between April–December 2021 in Italy. Viruses.

[14] Petrone D., Mateo-Urdiales A., Sacco C., Riccardo F., Bella A., Ambrosio L., Presti A.L., Di Martino A., Ceccarelli E., Del Manso M. (2023). Reduction of the risk of severe COVID-19 due to Omicron compared to Delta variant in Italy (November 2021–February 2022). Int. J. Infect. Dis..

[15] Nielsen R., Yang Z. (1998). Likelihood models for detecting positive selected amino acid sites and applications to the HIV-1 envelope gene. Genetics.

[16] Kosakovsky Pond S.L., Frost S.D.W. (2005). A genetic algorithm approach to detecting lineage-specific variation in selection pressure. Mol. Biol. Evol..

[17] European Centre for Disease Prevention and Control Public Health Impact of SARS-CoV-2 Variants of Concern: Scoping Review Protocol. https://www.ecdc.europa.eu/en/publications-data/public-health-impact-sars-cov-2-variants-concern-scoping-review-protocol.

[18] GISAID. https://gisaid.org/.

[19] Katoh K., Standley D.M. (2013). MAFFT Multiple Sequence Alignment Software Version 7: Improvements in Performance and Usability. Mol. Biol. Evol..

[20] Galaxy Platform. https://usegalaxy.org/.

[21] Afgan E., Baker D., Batut B., van den Beek M., Bouvier D., Čech M. (2018). The Galaxy platform for accessible, reproducible and collaborative biomedical analyses: 2018 update. Nucleic Acids Res..

[22] Hall T.A. (1999). BioEdit: A user-friendly biological sequence alignment editor and analysis program for windows 95/98/NT. Nucleic Acids Symp. Ser..

[23] Slatkin M., Maddison W.P. (1989). A cladistic measure of gene flow inferred from the phylogenies of alleles. Genetics.

[24] Nguyen L.-T., Schmidt H.A., von Haeseler A., Minh B.Q. (2015). IQ-TREE: A fast and effective stochastic algorithm for estimating maximum likelihood phylogenies. Mol. Biol. Evol..

[25] Minh B.Q., Nguyen M.A.T., von Haeseler A. (2013). Ultrafast approximation for phylogenetic bootstrap. Mol. Biol. Evol..

[26] Zhang J., Nielsen R., Yang Z. (2005). Evaluation of an improved branch-site likelihood method for detecting positive selection at the molecular level. Mol. Biol. Evol..

[27] Murrell B., Moola S., Mabona A., Weighill T., Sheward D., Kosakovsky Pond S.L., Scheffler K. (2013). FUBAR: A Fast, Unconstrained Bayesian AppRoximation for Inferring Selection. Mol. Biol. Evol..

[28] Kosakovsky Pond S.L., Frost S.D.W. (2005). Not So Different after All: A Comparison of Methods for Detecting Amino Acid Sites under Selection. Mol. Biol. Evol..

[29] Pond S.L.K., Frost S.D.W., Spencer V.M. (2005). HyPhy: Hypothesis testing using phylogenies. Bioinformatics.

[30] Ghosh N., Nandi S., Saha I. (2022). Phylogenetic analysis of 17271 Indian SARS-CoV-2 genomes to identify temporal and spatial hotspot mutations. PLoS ONE.

[31] WHO Weekly Epidemiological Update on COVID-19-30 March 2021. https://www.who.int/publications/m/item/weekly-epidemiological-update-on-covid-19---31-march-2021.

[32] Tian D., Sun Y., Zhou J., Ye Q. (2021). The Global Epidemic of the SARS-CoV-2 Delta Variant, Key Spike Mutations and Immune Escape. Front. Immunol..

[33] Chavda V.P., Bezbaruah R., Deka K., Nongrang L., Kalita T. (2022). The Delta and Omicron Variants of SARS-CoV-2: What We Know So Far. Vaccines.

[34] Stima della Prevalenza delle Varianti VOC (Variant Of Concern) e di altre varianti di SARS-CoV-2 in Italia.Quick Survey 17 January 2022. https://www.epicentro.iss.it/coronavirus/pdf/sars-cov-2-monitoraggio-varianti-indagini-rapide-17-gennaio-2022.pdf.

[35] Stima Della Prevalenza Delle Varianti VOC (Variant of Concern) e di Altre Varianti di SARS-CoV-2 in Italia. Quick Survey 31 January 2022. https://www.epicentro.iss.it/coronavirus/pdf/sars-cov-2-monitoraggio-varianti-indagini-rapide-31-gennaio-2022.pdf.

[36] Véras N.M.C., Santoro M.M., Gray R.R., Tatem A.J., Presti A.L., Olearo F., Cappelli G., Colizzi V., Takou D., Torimiro J. (2011). Molecular epidemiology of HIV type 1 CRF02_AG in Cameroon and African patients living in Italy. AIDS Res. Hum. Retrovir..

[37] Lo Presti A., Rezza G., Stefanelli P. (2020). Selective pressure on SARS-CoV-2 protein coding genes and glycosylation site prediction. Heliyon.

[38] Hodcroft E.B., Zuber M., Nadeau S., Vaughan T.G., Crawford K.H.D., Althaus C.L., Reichmuth M.L., Bowen J.E., Walls A.C., Corti D. (2021). Spread of a SARS-CoV-2 variant through Europe in the summer of 2020. Nature.

[39] Kannan S.R., Spratt A.N., Sharma K., Chand H.S., Byrareddy S.N., Singh K. (2022). Omicron SARS-CoV-2 variant: Unique features and their impact on pre-existing antibodies. J. Autoimmun..

[40] Saxena S.K., Kumar S., Ansari S., Paweska J.T., Maurya V.K., Tripathi A.K., Abdel-Moneim A.S. (2022). Characterization of the novel SARS-CoV-2 Omicron (B.1.1.529) variant of concern and its global perspective. J. Med. Virol..

[41] Magazine N., Zhang T., Wu Y., McGee M.C., Veggiani G., Huang W. (2022). Mutations and Evolution of the SARS-CoV-2 Spike Protein. Viruses.

[42] Upadhyay V., Lucas A., Panja S., Miyauchi R., Mallela K.M.G. (2021). Receptor binding, immune escape, and protein stability direct the natural selection of SARS-CoV-2 variants. J. Biol. Chem..

[43] Weisblum Y., Schmidt F., Zhang F., DaSilva J., Poston D., Lorenzi J.C., Muecksch F., Rutkowska M., Hoffmann H.-H., Michailidis E. (2020). Escape from neutralizing antibodies by SARS-CoV-2 spike protein variants. Elife.

[44] Liu Z., VanBlargan L.A., Bloyet L.-M., Rothlauf P.W., Chen R.E., Stumpf S., Zhao H., Errico J.M., Theel E.S., Liebeskind M.J. (2021). Identification of SARS-CoV-2 spike mutations that attenuate monoclonal and serum antibody neutralization. Cell Host Microbe.

[45] McCallum M., De Marco A., Lempp F.A., Tortorici M.A., Pinto D., Walls A.C., Beltramello M., Chen A., Liu Z., Zatta F. (2021). N-terminal domain antigenic mapping reveals a site of vulnerability for SARS-CoV-2. Cell.

[46] Shen L., Triche T.J., Bard J.D., Biegel J.A., Judkins A.R., Gai X. (2021). Spike Protein NTD mutation G142D in SARS-CoV-2 Delta VOC lineages is associated with frequent back mutations, increased viral loads, and immune evasion. medRxiv.

[47] COG UK-UK Data. https://sars2.cvr.gla.ac.uk/cog-uk/.

