# Supplementary material for: Tracking the Selective Pressure Profile and Gene Flow of SARS-CoV-2 Delta Variant in Italy from April to October 2021 and Frequencies of Key Mutations from Three Representative Italian Regions
Source: Microorganisms. 2023 Oct 27;11(11):2644. doi: 10.3390/microorganisms11112644 (PMC10673055; doi:10.3390/microorganisms11112644)
Supplement: Supplementary file 1 [file microorganisms-11-02644-s001.zip › Table S1.pdf]

Table S1. Selective pressure analysis on SARS-CoV-2 delta variant Italian protein coding sub-sets genomes

	nsp1
Positively selected sites (w for sites > 1)<br>HYPHY software	62 (P, S); 87 (E, D); 94 (G, S, V)
Negatively selected sites (w for sites < 1)<br>HYPHY software	16 (L), 35 (V), 105 (G), 116 (V), 160 (N)
	nsp2
Positively selected sites (w for sites > 1)<br>HYPHY software	27 (R,C); 81 (K,N), 89 (E,K), 129 (P,L,S), 155 (D, G), 159 (A, V), 263 (S, F), 318 (A, V), 339 (G,S), 485 (V, I), 496 (Q, P, H)
Negatively selected sites (w for sites < 1)<br>HYPHY software	8 (N); 20 (C); 101 (I); 154 (G); 179 (Y); 236 (C); 308 (V); 311 (V); 312 (K); 339 (G, S); 340 (A, V); 425 (V); 457 (E); 560 (I)
	nsp3
Positively selected sites (w for sites > 1)<br>HYPHY software	126 (S, L); 384 (K, N); 862 (L,F); 1228 (P, L); 1791 (L, F)
Negatively selected sites (w for sites < 1)<br>HYPHY software	9 (D), 12 (V), 21 (V), 75 (L), 89 (L), 119 (E), 237 (T), 249 (H), 363 (N), 390 (K), 438 (Y), 466 (P), 506 (N), 534 (A), 567 (T), 622 (D), 629 (T), 646 (R), 667 (A), 694 (K), 714 (D), 717 (V), 806 (D), 881 (Y), 884 (A), 891 (N), 918 (F), 952 (Y), 955 (T), 978 (Y), 988 (M, G), 989 (M, S), 1089 (F), 1095 (D), 1107 (F), 1117 (I), 1141 (F), 1159 (P), 1181 (N), 1188 (N), 1210 (L), 1224 (E), 1254 (V), 1377 (P), 1416 (L), 1520 (F), 1528 (I), 1622 (G), 1649 (D), 1672 (I), 1698 (H), 1730 (K), 1828 (V), 1846 (E), 1859 (Y), 1860 (N), 1885 (V).
	nsp4
Positively selected sites (w for sites > 1)	204 (T, I), 279 (D, N), 295 (T, I), 296 (C, F)

HYPHY software	
Negatively selected sites (w for sites < 1) HYPHY software	43 (I), 66 (N), 89 (P), 140 (I), 144 (D), 192 (V), 222 (R), 223 (H), 258 (V), 273 (Q), 290 (V), 318 (N), 337 (F), 378 (T), 390 (F), 410 (S), 416 (A), 443 (R), 445 (L)
	nsp5
Positively selected sites (w for sites > 1) HYPHY software	/
Negatively selected sites (w for sites < 1) HYPHY software	187 (D)
	nsp6
Positively selected sites (w for sites > 1) HYPHY software	2 (A, V), 6 (T, I), 27 (Q, R), 37 (L, F)
Negatively selected sites (w for sites < 1) HYPHY software	16 (L), 179 (V), 183 (M, I), 200 (F), 229 (C), 259 (L)
	nsp7
Positively selected sites (w for sites > 1) HYPHY software	/
Negatively selected sites (w for sites < 1) HYPHY software	9 (T)
	nsp8
Positively selected sites (w for sites > 1) HYPHY software	/
Negatively selected sites (w for sites < 1)	185 (I)

HYPHY software	
	nsp9
Positively selected sites (w for sites > 1)<br>HYPHY software	/
Negatively selected sites (w for sites < 1)<br>HYPHY software	112 (L)
	nsp10
Positively selected sites (w for sites > 1)<br>HYPHY software	/
Negatively selected sites (w for sites < 1)<br>HYPHY software	34 (G), 48 (H), 59 (P)
	nsp11
Positively selected sites (w for sites > 1)<br>HYPHY software	/
Negatively selected sites (w for sites < 1)<br>HYPHY software	/
	nsp12
Positively selected sites (w for sites > 1)<br>HYPHY software	46 (A, S), 61 (E, D, K), 95 (A, S), 141 (T, I), 197 (R, Q), 323 (P, L), 384 (S, P), 463 (M, I), 822 (Q, H), 838 (L, I)
Negatively selected sites (w for sites < 1)<br>HYPHY software	37 (I), 39 (N), 59 (K,N), 61 (E,D,K), 83 (E), 168 (N), 177 (N,K), 212 (L), 217 (Y), 221 (D), 235 (D), 273 (Y), 275 (F), 291 (D), 301 (C), 308 (L), 326 (F), 340 (F), 369 (K), 428 (F), 450 (I), 458 (Y), 463 (M, I), 495 (V), 541 (Q), 569 (R), 599 (H), 606 (Y), 608 (D), 657 (N), 691 (N), 700 (V), 701 (T), 702 (A), 713 (N), 752 (H), 760 (D), 765 (C), 769 (T), 770 (Y), 872 (H), 875 (Q), 885 (L), 891 (L), 894 (E), 910 (D).
	nsp13

Positively selected sites (w for sites > 1) HYPHY software	77 (P, L), 89 (V, I)
Negatively selected sites (w for sites < 1) HYPHY software	32 (D), 34 (V), 47 (P, L), 60 (V), 75 (H), 93 (Y), 139 (K), 151 (I), 177 (N), 234 (P), 258 (I), 311 (H), 318 (C), 323 (K), 336 (A), 360 (V), 401 (D), 420 (E), 446 (A, P), 462 (K), 521 (V)
	nsp14
Positively selected sites (w for sites > 1) HYPHY software	46 (P, L), 289 (R, H), 374 (S, F), 394 (A, V)
Negatively selected sites (w for sites < 1) HYPHY software	93 (G), 103 (T), 156 (G), 182 (V), 212 (R), 224 (Y), 232 (G), 235 (Y), 279 (C), 382 (C), 383 (L), 402 (D), 410 (N), 424 (H) , 428 (T)
	nsp15
Positively selected sites (w for sites > 1) HYPHY software	80 (A, V), 81 (A, V)
Negatively selected sites (w for sites < 1) HYPHY software	39 (D), 45 (N), 84 (V), 85 (I), 116 (C), 137 (A), 145 (E)
	nsp16
Positively selected sites (w for sites > 1) HYPHY software	9 (V, I), 34 (A, V), 215 (P, L, T)
Negatively selected sites (w for sites < 1) HYPHY software	15 (Y), 17 (M, I), 45 (A), 55 (N), 56 (T), 66 (R), 93 (T), 107 (A), 116 (A), 155 (G), 167 (V), 170 (K), 199 (A), 237 (I), 250 (F), 266 (Q), 277 (K)
	spike
Positively selected sites (w for sites > 1)	5 (L, F), 70 (V, I, F), 95 (T, I), 142 (G, D, Y, H, V), 222 (A, S, V), **367 (V, L, H, F), 452 (L, R), 501 (N, Y),** 613 (Q, H), 614 (D, G), 677 (Q, H), 681 (P,R),

HYPHY software	950 (D, N), 1104 (V, L), 1128 (V, L), 1219 (G, V, C)
Negatively selected sites (w for sites < 1) HYPHY software	32 (F), 102 (R), 106 (F), 137 (N, K), 234 (N), 296 (L), 302 (T), 312 (I), 346 (R), 395 (V), 400 (F), 410 (I), 432 (C), 477 (S), 603 (N), 649 (C), 665 (P), 707 (Y), 732 (T), 760 (C), 856 (N), 935 (Q), 1061 (V), 1065 (V), 1119 (N), 1122 (V), 1230 (V), 1239 (S), 1257 (D)
	ORF3a
Positively selected sites (w for sites > 1) HYPHY software	41 (L, I, F), 110 (A, S, V)
Negatively selected sites (w for sites < 1) HYPHY software	22 (D), 101 (L)
	E
Positively selected sites (w for sites > 1) HYPHY software	/
Negatively selected sites (w for sites < 1) HYPHY software	/
	M
Positively selected sites (w for sites > 1) HYPHY software	82 (I, T)
Negatively selected sites (w for sites < 1) HYPHY software	53 (F), 88 (V), 121 (N), 203 (N)
	ORF6

Positively selected sites (w for sites > 1)<br>HYPHY software	/
Negatively selected sites (w for sites < 1)<br>HYPHY software	16 (L)
	ORF7a
Positively selected sites (w for sites > 1)<br>HYPHY software	/
Negatively selected sites (w for sites < 1)<br>HYPHY software	69 (D)
	ORF8
Positively selected sites (w for sites > 1)<br>HYPHY software	/
Negatively selected sites (w for sites < 1)<br>HYPHY software	46 (Y)
	N
Positively selected sites (w for sites > 1)<br>HYPHY software	9 (Q, L, H)
Negatively selected sites (w for sites < 1)<br>HYPHY software	13 (P), 35 (A), 110 (F), 128 (D), 154 (N), 158 (V), 297 (D), 298 (Y), 302 (P), 329 (T)
	ORF 10
Positively selected sites (w for sites > 1)<br>HYPHY software	/

Negatively selected sites (w for sites < 1)	/

HYPHY software
