# Supplementary material for: Tracking the Selective Pressure Profile and Gene Flow of SARS-CoV-2 Delta Variant in Italy from April to October 2021 and Frequencies of Key Mutations from Three Representative Italian Regions
Source: Microorganisms. 2023 Oct 27;11(11):2644. doi: 10.3390/microorganisms11112644 (PMC10673055; doi:10.3390/microorganisms11112644)
Supplement: Supplementary file 1 [file microorganisms-11-02644-s001.zip › Table S2.pdf]

Table S2. The prediction of the possible impact of the amino acid substitutions on the stability and structure of the proteins through I-Mutant 2.0 and PolyPhen-2.

		PolyPhen - 2		I- Mutant 2.0	
**Mutation**	**Target**	**Prediction**	**Score**	**Stability**	**DDG**
P62S	nsp1	Probably Damaging	0.978	Decrease	-1.84
E87D	nsp1	Probably Damaging	0.994	Decrease	-0.11
G94S	nsp1	Probably Damaging	1	Decrease	-0.71
G94V	nsp1	Probably Damaging	1	Decrease	-0.19
R27C	nsp2	Probably Damaging	1	Decrease	-0.35
K81N	nsp2	Probably Damaging	1	Increase	0.05
E89K	nsp2	Probably Damaging	0.995	Decrease	-1.34
P129L	nsp2	Possibly Damaging	0.924	Decrease	-0.53
P129S	nsp2	Probably Damaging	1	Decrease	-2.45
D155G	nsp2	Probably Damaging	0.997	Increase	0.09
A159V	nsp2	Probably Damaging	0.999	Decrease	-0.25
S263F	nsp2	Possibly Damaging	0.528	Increase	0.34
A318V	nsp2	Benign	0.043	Decrease	-1.24
G339S	nsp2	Probably Damaging	1	Decrease	-1.57
V485I	nsp2	Benign	0.092	Increase	0.33
Q496P	nsp2	Possibly Damaging	0.607	Decrease	-2.48
Q496H	nsp2	Benign	0.003	Decrease	-1.69
S126L	nsp3	Possibly Damaging	0.585	Increase	0.94
K384N	nsp3	Benign	0.001	No Change	0.00
L862F	nsp3	Probably Damaging	1	Decrease	-0.52
P1228L	nsp3	Benign	0.001	Decrease	-0.46
L1791F	nsp3	Probably Damaging	0.999	Decrease	-0.55
T204I	nsp4	Benign	0.002	Decrease	-1.08
D279N	nsp4	Probably Damaging	0.999	Decrease	-1.26
T295I	nsp4	Probably Damaging	0.999	Decrease	-0.02
C296F	nsp4	Possibly Damaging	0.896	Decrease	-0.70
A2V	nsp6	Benign	0.306	Decrease	-0.50
T6I	nsp6	Benign	0.000	Decrease	-0.93
Q27R	nsp6	Probably Damaging	0.998	Decrease	-0.95
L37F	nsp6	Benign	0.027	Decrease	-0.05
A46S	nsp12	Benign	0.001	Decrease	-0.64
E61D	nsp12	Benign	0.000	Decrease	-0.86
E61K	nsp12	Benign	0.159	Decrease	-1.74
A95S	nsp12	Benign	0.090	Decrease	-0.55
T141I	nsp12	Benign	0.008	Decrease	-1.65
R197Q	nsp12	Benign	0.211	Decrease	-0.7
P323L	nsp12	Benign	0.018	Decrease	-0.8

		PolyPhen - 2		I- Mutant 2.0	
**Mutation**	**Target**	**Prediction**	**Score**	**Stability**	**DDG**
S384P	nsp12	Possibly Damaging	0.715	Decrease	-0.07
M463I	nsp12	Possibly Damaging	0.929	Decrease	-0.55
Q822H	nsp12	Benign	0.000	Decrease	-1.29
L838I	nsp12	Probably Damaging	0.998	Decrease	-1.61
P77L	nsp13	Probably Damaging	1	Decrease	-1.03
V89I	nsp13	Benign	0.103	Decrease	-0.78
P46L	nsp14	Possibly Damaging	0.557	Decrease	-1.39
R289H	nsp14	Possibly Damaging	0.860	Decrease	-2.11
S374F	nsp14	Benign	0.001	Decrease	-0.16
A394V	nsp14	Benign	0.005	Decrease	-0.17
A80V	nsp15	Benign	0.002	Decrease	-0.66
A81V	nsp15	Probably Damaging	0.988	Decrease	-0.43
V9I	nsp16	Benign	0.018	Increase	0.31
A34V	nsp16	Benign	0.018	Decrease	-1.3
P215L	nsp16	Benign	0.000	Decrease	-1.68
P215T	nsp16	Benign	0.000	Decrease	-1.92
L5F	spike	Unknown	not available	Decrease	-0.10
V70I	spike	Benign	0.000	Decrease	-1.58
V70F	spike	Benign	0.066	Decrease	-2.72
T95I	spike	Probably Damaging	0.999	Decrease	-1.8
G142D	spike	Benign	0.03	Decrease	-1.17
G142Y	spike	Benign	0.278	Decrease	-0.24
G142H	spike	Possibly Damaging	0.867	Decrease	-1.57
G142V	spike	Benign	0	Decrease	-0.48
A222V	spike	Benign	0	Increase	0.48
A222S	spike	Benign	0.001	Decrease	-0.68
V367L	spike	Possibly Damaging	0.852	Decrease	-1.06
V367H	spike	Probably Damaging	1	Decrease	-2.32
V367F	spike	Possibly Damaging	0.852	Decrease	-3.08
L452R	spike	Benign	0.017	Decrease	-1.40
Q613H	spike	Probably Damaging	0.988	Decrease	-1.47
N501Y	spike	Benign	0.206	Decrease	-0.34
D614G	spike	Benign	0.009	Decrease	-1.94
Q677H	spike	Benign	0.157	Increase	0.10
P681R	spike	Unknown	not available	Decrease	-0.79
D950N	spike	Benign	0.340	Increase	0.15
V1104L	spike	Benign	0.001	Decrease	-0.09
V1128L	spike	Possibly Damaging	0.900	Decrease	-1.14
G1219V	spike	Probably Damaging	0.998	Decrease	-1.76
G1219C	spike	Probably Damaging	1	Decrease	-1.81

		PolyPhen - 2		I- Mutant 2.0	
**Mutation**	**Target**	**Prediction**	**Score**	**Stability**	**DDG**
L41F	ORF3a	Benign	0.006	Decrease	-1.36
L41I	ORF3a	Benign	0.286	Decrease	-0.93
A110S	ORF3a	Possibly Damaging	0.898	Decrease	-1.01
A110V	ORF3a	Probably Damaging	0.998	Increase	0.91
I82T	M	Possibly Damaging	0.889	Decrease	-2.41
Q9L	N	Benign	0.191	Increase	0.31
Q9H	N	Possibly Damaging	0.803	Decrease	-0.39
