# Supplementary material for: Tracking the Selective Pressure Profile and Gene Flow of SARS-CoV-2 Delta Variant in Italy from April to October 2021 and Frequencies of Key Mutations from Three Representative Italian Regions
Source: Microorganisms. 2023 Oct 27;11(11):2644. doi: 10.3390/microorganisms11112644 (PMC10673055; doi:10.3390/microorganisms11112644)
Supplement: Supplementary file 1 [file microorganisms-11-02644-s001.zip › Table S3.pdf]

Table S3. Significance identified for each amino acid mutation in the comparison of the data among the three regions.

Mutation	Lazio - Sicily	Lazio - Veneto	Veneto - Sicily
P62S	n.s.	n.s.	n.s.
E87D	n.s.	s.	s.
G94S	s.	s.	s.
G94V	n.s.	/	n.s.
R27C	/	/	/
K81N	s.	n.s.	s.
E89K	s.	/	s.
P129L	n.s.	n.s.	n.s.
P129S	/	/	/
D155G	n.s.	n.s.	n.s.
A159V	/	/	/
S263F	s.	n.s.	s.
A318V	s.	n.s.	s.
G339S	n.s.	n.s.	n.s.
V485I	n.s.	n.s.	n.s.
Q496P	n.s.	n.s.	n.s.
Q496H	/	/	/
S126L	/	n.s.	n.s.
K384N	n.s.	n.s.	n.s.
L862F	/	/	/
P1228L	s.	n.s.	s.
L1791F	/	n.s.	n.s.
T204I	n.s.	n.s.	n.s.
D279N	/	/	/
T295I	s.	/	s.
C296F	n.s.	n.s.	n.s.
A2V	s.	n.s.	s.
T6I	s.	s.	/
Q27R	/	s.	s.
L37F	s.	s.	n.s.
A46S	s.	/	s.
E61D	s.	/	s.
E61K	/	/	/
A95S	n.s.	n.s.	/
T141I	/	/	/
R197Q	s.	n.s.	s.
P323L	s.	/	s.

Mutation	Lazio - Sicily	Lazio - Veneto	Veneto - Sicily
S384P	n.s.	n.s.	n.s.
M463I	n.s.	n.s.	n.s.
Q822H	s.	s.	s.
L838I	s.	n.s.	s.
P77L	/	s.	s.
V89I	/	/	/
P46L	n.s.	n.s.	n.s.
R289H	s.	s.	n.s.
S374F	n.s.	n.s.	n.s.
A394V	s.	n.s.	s.
A80V	s.	/	n.s.
A81V	n.s.	n.s.	/
V9I	/	/	/
A34V	n.s.	s.	n.s.
P215L	n.s.	n.s.	/
P215T	n.s.	n.s.	n.s.
L5F	n.s.	n.s.	n.s.
V70I	/	/	/
V70F	/	/	/
T95I	s.	n.s.	s.
G142D	s.	s.	s.
G142Y	/	/	/
G142H	/	/	/
G142V	/	/	/
A222V	s.	n.s.	s.
A222S	/	/	/
V367L	/	/	/
V367H	/	/	/
V367F	/	/	/
L452R	s.	s.	/
Q613H	s.	n.s.	s.
N501Y	n.s.	/	n.s.
D614G	/	/	/
Q677H	s.	ns	s.
P681R	/	/	/
D950N	s.	s.	s.
V1104L	n.s.	n.s.	n.s.
V1128L	/	/	/
G1219V	n.s.	/	n.s.
G1219C	/	/	/
L41F	n.s.	n.s.	n.s.

Mutation	Lazio - Sicily	Lazio - Veneto	Veneto - Sicily
L41I	/	/	/
A110S	s.	n.s.	s.
A110V	/	/	/
I82T	/	/	/
Q9L	s.	n.s.	s.
Q9H	/	/	/

n.s. not significant
s. significant
